# Longitudinal transition of body mass index status and its associated factors among Chinese middle-aged and older adults in Markov model

**DOI:** 10.3389/fpubh.2022.973191

**Published:** 2022-08-04

**Authors:** Heming Pei, Ning Kang, Chao Guo, Yalu Zhang, Haitao Chu, Gong Chen, Lei Zhang

**Affiliations:** ^1^Institute of Population Research, Peking University, Beijing, China; ^2^Division of Biostatistics, School of Public Health, University of Minnesota Twin Cities, Minneapolis, MN, United States; ^3^Institute of Ageing and Development, Peking University, Beijing, China

**Keywords:** body mass index, cohort study (or longitudinal study), multistate model, weight change, transition intensity

## Abstract

**Introduction:**

Body mass index (BMI) has a strong correlation with chronic diseases and all-cause mortality. However, few studies have previously reported the longitudinal transition of BMI status and its influential factors, especially among Chinese middle-aged and older adults.

**Methods:**

This population-based cohort study involved 6,507 participants derived from the China Health and Retirement Longitudinal Study from 2011 to 2015, including objectively measured BMI recorded in 26,028 person-year of all observations followed up. Multistate Markov model was performed to estimate the BMI state transition intensity and hazard ratios of each potential exposure risk.

**Results:**

The mean intensity of the population that shifted from normal to overweight was more than twice than shifted to underweight. Besides, a predicted probability was up to 16.16% that the population with overweight would suffer from obesity and more than half of the population with underweight would return to normal weight over a 6-year interval. The study also implied significant effects of baseline age, gender, marital status, education level, alcohol consumption, smoking, depression symptoms, and activities of daily living impairment on BMI status transition to varying degrees.

**Conclusions:**

Findings of this study indicated that the mean transition probability between different BMI statuses varied, specific exposure factors serving as barriers or motivators to future transitions based on current BMI status was clarified for the health promotion strategies.

## Introduction

Being underweight, overweight, or obese was associated with multiple adverse health consequences throughout the life course. Among the 84 risk factors evaluated by the Global Burden of Disease Study in 2017, high body mass index (BMI), by far, was with the greatest relative increase in exposure since 1990, and was among the leading five risk factors ranked by risk-attributable disability-adjusted life years (DALYs) (1). Higher BMI was associated with greater mortality from cardiovascular diseases (CVDs) to all causes of mortality (2–5). On the other hand, underweight was an explicit risk factor of self-rated health, cognition and low health-related quality of life (HRQoL) (6, 7). Stable BMI could act as a predictor of a lower risk of cardiovascular disease and premature death among individuals with insulin-treated diabetes (5).

Previous empirical evidence from the Non-communicable Diseases Risk Factor Collaboration of East Asia showed that the transition from underweight to overweight and obesity could be rapid and might overwhelm the national capacity needed to engender a healthy transition (8). According to the global epidemic study of obesity, China and other East Asian countries were at the stage featuring a smaller increase in the prevalence of obesity among children but a large increase among adults (9). During the past few decades, transitions in the structure of the population have led to a sharp increase in the number of middle-aged and older adults in China (10). In addition, Chinese elderly persons manifested the problems of over- and undernutrition, with 33% of women and 27% of men aged 60 years or older being overweight (11). This indicated that overweight and obesity represented one of the most burdensome and intractable public health issues at present.

Although studies of BMI change were numerous, most of the relevant research could be confounded by multifaceted methodological problems. Regardless of individual heterogeneity, using subjective judgment to divide study objectives into “weight gain” and “weight loss” groups as a frequently used criterion had led to a lack of accuracy in weight status identification. Furthermore, BMI acted as a time-varying variable so that classical forms of regression analyses were unable to identify the longitudinal transition process and generate causal inferences (12). In addition, the hierarchical modeling approaches developed in recent years such as group-based trajectory models or latent growth curve models handled the weight status with BMI as a continuous variable where linearity assumption was made with the limitation that the threshold effect of BMI on weight status was neglected.

On the other hand, longitudinal data often consisted of observations throughout the changing process at arbitrary times, so the exact times when the state change occurred were inaccessible. As sampling times in panel data were assumed to be non-informative for the conventional survival analyses framework, such as the Kaplan Meier or Cox proportional regression, a continuous-time Markov process considering individual heterogeneity appeared as an appropriate alternative method, which meant the BMI status of a participant could change at any time and even more than once within each survey interval (13). This proved to be a useful way of simulating a process in which an individual moved through a series of states continuously, and it had been used for a wide range of medical situations, including problems following heart transplantation (14), hepatic cancer (15), HIV infection and AIDS (16) and diabetes complications (17). With prerequisites of the Markov process precisely met, the BMI status transition process was depicted by transition intensity and probability through a multistate Markov model.

This study aimed to investigate the longitudinal transition of BMI status among Chinese middle-aged and older adults and the effects of various exposure factors on the likelihood of BMI status transition using a multistate Markov model to provide insights into underlying determinants and future trends.

## Materials and methods

### Data collection

The data used in this study were derived from three waves (2011, 2013, and 2015) of the China Health and Retirement Longitudinal Study (CHARLS) (5). CHARLS provided nationally representative panel data enable inferences to be made about the social, economic, and health circumstances of the Chinese population aged 45 years or older.

The national baseline survey for the study was conducted between June 2011 and March 2012 including participants living in 10,257 households in 450 villages or urban communities. A stratified (by per capita GDP of urban districts and rural counties), multistage (county, district, village, community, and household), probability proportional to size random sampling strategy was adopted (11). With adjusted weights, physical examinations were performed with 13,974 participants at baseline.

All participants were eligible for inclusion if they satisfied the following criteria: (a) had 4 years of follow-up, (b) biomarkers of anthropometrics for each survey were fully recorded and no outliers (such as BMI <15.0 or equal to or >60.0) existed, and (c) baseline age was 45 years or older. The statistical analyses featured data on 6,507 participants. Multivariate logistic regression was performed to evaluate selection bias, as the results indicated that sampling had no significant difference on distributions of demographic characteristics between exclusive and inclusive sample (age: β = 0.002; gender: β = −0.030; education: β = −0.12/0.20) which implied that the samples included in the final analysis was representative enough.

Ethical approval for all study waves was granted by the Institutional Review Board at Peking University. The approval number for the main household survey, including anthropometrics, is IRB00001052-11015; the approval number for-6 biomarker collection is IRB00001052-11014.

### Assessment of BMI status

Biomarkers were collected in 2011, 2013, and 2015, and clinical physical measurements were conducted with unified equipment that enabled the longitudinal transition of BMI status to be monitored accurately. Weight was assessed without shoes and with lightweight clothing to the nearest 0.1 kg using the Omron HN-286 scale, and height was measured to the nearest 0.1 cm without shoes using the portable SECA 213 stadiometer. BMI was calculated as kilograms per meter squared, and the BMI status was categorized as underweight (BMI <18.5), normal weight (18.5 ≤ BMI <24), overweight (24 ≤ BMI <28), and obese (BMI ≥ 28), which aligns with the criteria for adult weight (Chinese standard, WS/T 428-2013) (18).

### Measurement of exposure factors

To estimate the effect of potential risk factors, demographic and health status variables were obtained from a structured questionnaire, including age, gender, marital status, education level, behavioral factors (alcohol consumption, smoking), depression symptoms, activities of daily living (ADL), and blood pressure status with objective measures, which were selected based on previous studies (19, 20). Variables were formatted to be consistent across studies based on standard categories: Age was grouped as the World Health Organization age classification criteria (45–59, 60–74, ≥ 75 years); alcohol consumption was converted into an binary variable (drinking less than once a month vs. drink more than once a month); blood pressure was assessed three times at 45 s intervals with an Omron HEM-7,200 monitor (hypertension was diagnosed as systolic pressure ≥ 140 mmHg [1 mmHg = 0.133 kPa] or diastolic pressure ≥ 90 mmHg); depression was measured by the 10-item Center for Epidemiological Studies Depression Scale using a cutoff score of 10 or higher as the indicator of depression symptoms (21); and the Katz ADL Index summarized overall functional performance in bathing, dressing, going to the toilet, transferring, continence, and feeding to evaluate independence in ADL and was dichotomized as ADL independent (total score = 12) and ADL impairment (total score <12) with two points for each item (22). The reliability and validity of these measures (Chinese version) among middle-aged and older adults have been previously established (23). Other individual medical history (diabetes, chronic lung diseases, Cardiovascular diseases, digestive diseases), which were self-reported by the participants, all were based on clinical diagnoses in secondary or higher hospitals.

### Statistical analysis

Baseline characteristics of the participants were summarized in Table 1. Categorical data were presented as percentages (with sample size) and grouped by BMI status occupied at baseline.

**Table 1:** Baseline characteristics of the participants under study.

**Characteristic**	**Total (*N* = 6,507)**	**Underweight (*n* = 425)**	**Normal (*n* = 3,453)**	**Overweight (*n* = 1,894)**	**Obesity (*n* = 735)**
**Age**
45–59	3,537 (54.4)	143 (33.6)	1,786 (51.7)	1,117 (59.0)	491 (66.8)
60–74	2,630 (40.4)	221 (52.0)	1,472 (42.6)	708 (37.4)	229 (31.2)
≥75	340 (5.2)	61 (14.4)	195 (5.7)	69 (3.6)	15 (2.0)
**Gender**
Male	3,059 (47.0)	199 (46.8)	1,844 (53.4)	772 (40.8)	244 (33.2)
Female	3,448 (53.0)	226 (53.2)	1,609 (46.6)	1,122 (59.2)	491 (66.8)
**Marital status**
Married with spouse present	5,533 (85.0)	335 (78.8)	2,882 (83.5)	1,673 (88.3)	643 (87.5)
Others^*^	974 (15.0)	90 (21.2)	571 (16.5)	221 (11.7)	92 (12.5)
**Education level**
Illiterate	1,781 (27.4)	158 (37.2)	946 (27.4)	477 (25.2)	200 (27.2)
Elementary school	2,795 (43.0)	195 (45.9)	1,543 (44.7)	771 (40.7)	286 (38.9)
Middle school and above	1,931 (29.7)	72 (16.9)	964 (27.9)	646 (34.1)	249 (33.9)
High blood pressure	632 (9.7)	24 (5.7)	236 (6.8)	238 (12.6)	134 (18.2)
Diabetes	348 (5.4)	6 (1.4)	112 (3.2)	147 (7.8)	83 (11.3)
Chronic lung diseases	654 (10.1)	81 (19.1)	351 (10.2)	165 (8.7)	57 (7.8)
Cardiovascular diseases	733 (11.3)	44 (10.4)	308 (8.9)	249 (13.2)	132 (18.0)
Digestive diseases	1,505 (23.1)	127 (29.9)	814 (23.6)	430 (22.7)	134 (18.2)
**Alcohol consumption**
Drink but less than once a month	4,919 (75.6)	339 (79.8)	2,475 (71.7)	1,484 (78.4)	621 (84.5)
Drink more than once a month	1,588 (24.4)	86 (20.2)	978 (28.3)	410 (21.6)	114 (15.5)
Smoking	2,542 (39.0)	192 (45.2)	1,557 (45.1)	602 (31.8)	191 (26.0)
Depression symptoms	2,465 (37.9)	225 (52.9)	1,340 (38.8)	644 (34.0)	256 (34.8)
ADL impairment^†^	222 (3.4)	23 (5.4)	105 (3.0)	60 (3.2)	34 (4.6)

* Others included single, divorced and widowed.

† ADL, activities of daily living.

The Markov assumption was that future evolution only depends on the current state, which was highly consistent with the general patterns of BMI status change. For BMI as a continuous variable, the transitions in BMI status were permitted to occur between neighboring states: underweight to normal, normal to overweight, and overweight to obese. That is, at next observation window, BMI status could only shift to an adjacent state.

**Figure 1:**
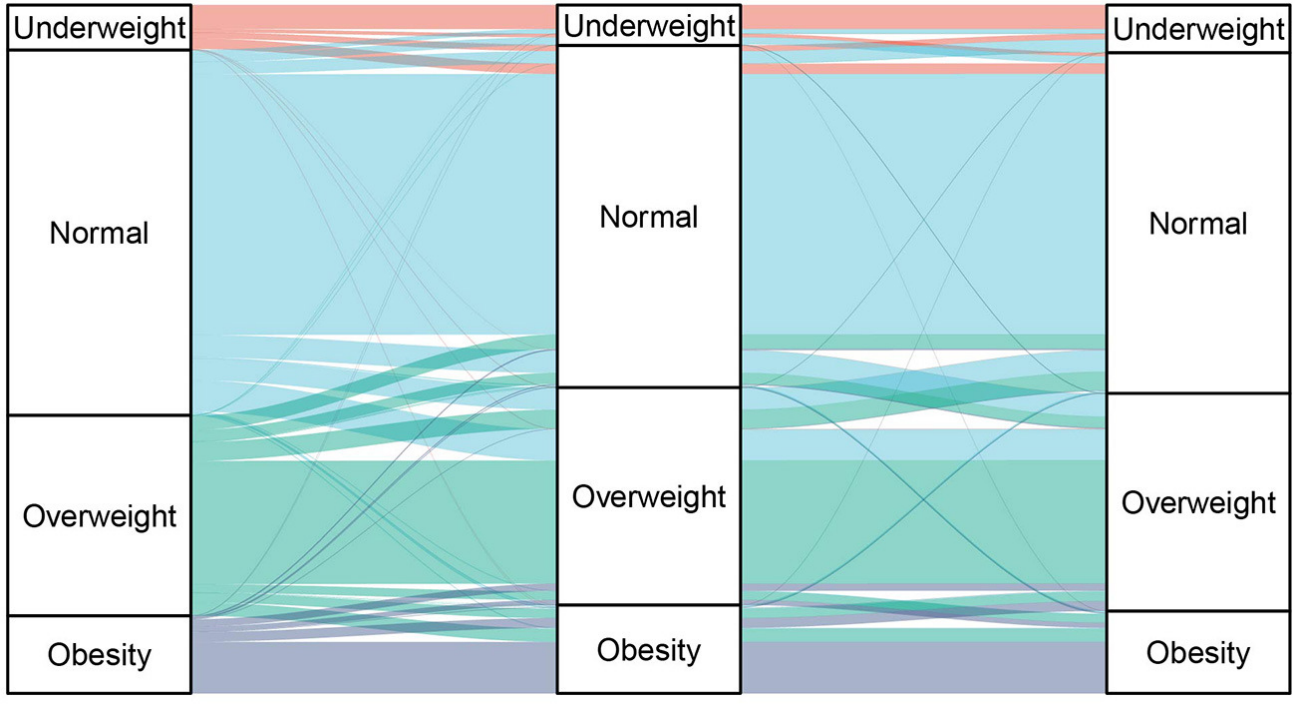
Longitudinal transition on BMI status among Chinese middle aged and older adults across three waves.

**Figure 2:**
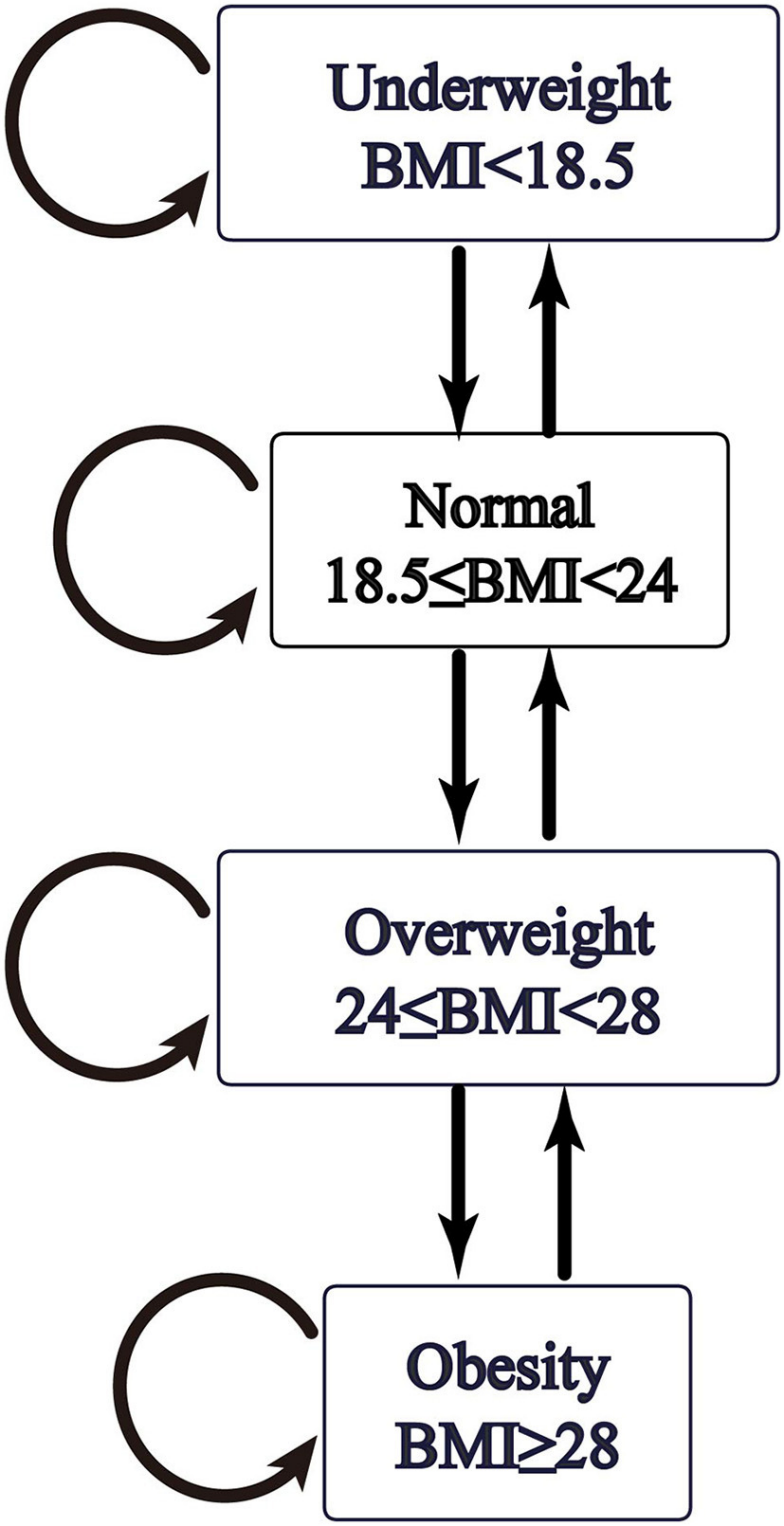
Hypothesized transition diagram of Markov process based on BMI status change.

As the prior plausibility of the transition pattern was verified in Figure 1, the hypothesized transition of BMI states was specified by arrows in Figure 2. The subsequent calculation of intensity and probability would be analytically based on initial transition structure illustrated in the model diagram.

To summary the multistate data, a frequency table was produced with pairs of consecutive states covering all individuals corresponded to the number of occasions for each state transition (Table 2). The next state to where the individual moved was governed by a transition intensity matrix *Q*. Although panel data was considered “snapshots” of the process, the intensity represented the instantaneous risk of moving from one state to another. As in the theoretical model illustrated by Figure 2, fitting the model was a process of finding values of the six unknown parameters of transition intensity to specify a Markov chain:


$$\begin{array}{l} \text{Q}=\left(\begin{array}{c} \begin{array}{cccc} -{\text{q}}_{12} & {\text{q}}_{12} & 0 & 0\\ {\text{q}}_{21} & -\left({\text{q}}_{21}+{\text{q}}_{23}\right) & {\text{q}}_{23} & 0\\ 0 & {\text{q}}_{32} & -\left({\text{q}}_{32}+{\text{q}}_{34}\right) & {\text{q}}_{34}\\ 0 & 0 & {\text{q}}_{43} & -{\text{q}}_{43} \end{array} \end{array}\right) \end{array}$$

**Table 2:** BMI transition frequency and estimated transition intensity matrix.

	**State**_**T+1**_ ^†^							
State${}_{\text{T}}^{*}$	**Underweight**		**Normal**		**Overweight**		**Obesity**	
	**Frequency**	**Intensity (95%CI)**	**Frequency**	**Intensity (95%CI)**	**Frequency**	**Intensity (95%CI)**	**Frequency**	**Intensity (95%CI)**
Underweight	535	−0.21 (-0.24,−0.19)	254	0.21 (0.19, 0.24)	13	–	5	–
Normal	286	0.03 (0.03, 0.03)	5,512	−0.11 (-0.12,−0.10)	833	0.08 (0.08, 0.09)	55	–
Overweight	8	-	636	0.11 (0.10, 0.11)	2,954	−0.17 (-0.18,−0.16)	352	0.06 (0.06, 0.07)
Obesity	4	–	51	–	312	0.14 (0.13, 0.16)	1,204	−0.14 (-0.16,−0.13)

The BMI transition frequency and transition intensity matrix were estimated by bootstrapping over 1000 iterations from rows to columns.

^*^State_T_ : BMI state in current occasion.

† State_T+1_: BMI state in next occasion.

As shown here, all allowed transitions of our model were defined with the initial value in each position. Any diagonal entry in the matrix was ignored, because it was constrained to be equal to the negative sum of the rest of the row. The maximum likelihood estimated transition intensity matrix and 95% confidence intervals by bootstrap resampling were extracted *via* the multistate Markov model, as shown in Table 2. Rows corresponded to the “from state” and columns to the “to state” with transition intensities estimated on the log scale.

For a continuous-time homogeneous process, a fitted continuous multistate model was also assessed to predict the transition probabilities for different intervals (24). The probability of conditionally entering a given state during a period *t* was calculated by taking the matrix exponential of the scaled transition intensity matrix *Q*.


$$\begin{array}{l} {\text{P}}_{\left(\text{t}\right)}=\text{Exp}\left(\text{tQ}\right) \end{array}$$

Transition intensities might also depend on a series of individual-specific variables. The relation between individual characteristics and their transition rate was assessed by a multistate model. Explanatory variables including age, gender, marital status, education level, blood pressure, alcohol consumption, smoking, depression symptoms, and ADL impairment for a certain type of transition could be investigated by modeling the intensity as a function of these variables. Four BMI statuses through which the participants could move were labeled as State 1, State 2, State 3, and State 4; hazard ratios of each exposure effect were computed by exponentiating the estimated covariate effects on the log transition intensities, and an approximate upper and lower confidence limit was also calculated with assuming normality of the log effect with 95% confidence intervals (13).

All analyses were performed using the statistical software R version 6.1. The R codes for our analyses are available upon request.

## Results

### Baseline characteristics of study participants

The 6,507 participants who completed the survey during a 4-year follow-up provided a diversity in sociodemographic features and BMI statuses. Participants with normal (53.1%) and overweight (29.1%) BMI status comprised majority of the population. The distribution of demographic variables was even, such as the age (45–59 years: 54.4%; 60–74 years: 40.4%; ≥ 75 years: 5.2%; Kolmogorov-Smirnov test *p* = 0.98) and the gender (male: 53.0%; female: 47.0%). The proportion of illiterate (37.2%) and other marital status (21.2%) groups was highest among those in underweight status. Compared with participants in normal BMI status, the prevalence of high blood pressure and ADL impairment increased with a higher BMI in this study (*p* < 0.01 in chi-square test for trend in proportions).

### The longitudinal transition between the BMI statuses

The longitudinal transition between BMI statuses across three observation occasions among Chinese middle-aged and older adults by Sankey diagram was depicted in Figure 1. Although the overall distribution of BMI status remained stable, transitions occurred between adjacent states in each 2-year interval. Abrupt changes were negligible, as shown in Figure 1, where the practical dynamics situation assumed the Markov process of BMI status change and determined initial transition intensity matrix.

Among 26,028 person-year follow-up observations, the transition frequency during the study and transition intensity matrix calculated in a multistate Markov model by bootstrapping with 1,000 iterations (from rows to columns) as printed in Table 2; cumulative BMI status observations reflected that most transitions occurred between normal weight and overweight (833 + 636), and shifts from overweight to obesity (352) were also considerable among these Chinese middle-aged and older adults. As for the transition intensity matrix, the results indicate that the mean intensity of shifting from normal BMI to overweight was more than twice that of shifting from normal to underweight (0.08/0.03 ≈ 2.67); in parallel, the mean intensity of shifting from overweight to normal BMI was nearly twice the intensity of shifting from overweight to obesity (0.11/0.06 ≈ 1.83). The other estimates in Table 2 could be interpreted in the same manner. Moreover, the diagonal elements of the *r*th in the estimated transition intensity matrix could also reflect the mean sojourn times in each transient state as−1/q_rr_, where the state of normal weight had the longest period of occupancy with a mean of 9.12 years [-1/(-0.11)] and the underweight state had the shortest sojourn time of 4.73 years [-1/(-0.21)].

Table 3 presented estimates based on a multistate Markov model that predicted the transition probabilities of conditionally entering a state over a series of intervals of 2, 4, and 6 years. The probability that an individual would move from normal weight to overweight was 12.49% and 19.85% in 2 and 4 years, respectively; the estimated probability that an individual with overweight would progress to obesity was 16.16%, whereas more than half (53.83%) of the population with underweight recovering back to normal weight status required a 6-year interval.

**Table 3:** Estimate of the transition probability among different BMI states during follow-up time intervals^*^ (%).

**Time/State**	**2 years follow-up**				**4 years follow-up**				**6 years follow-up**			
	**Underweight**	**Normal**	**Overweight**	**Obesity**	**Underweight**	**Normal**	**Overweight**	**Obesity**	**Underweight**	**Normal**	**Overweight**	**Obesity**
Underweight	66.33	31.07	-	-	45.31	46.67	-	-	32.04	53.83	-	-
Normal	4.19	82.56	12.49	-	6.29	71.51	19.85	-	7.26	64.28	24.31	-
Overweight	-	16.29	74.17	9.10	-	25.87	58.98	13.84	-	31.69	49.93	16.16
Obesity	-	-	21.12	76.54	-	-	32.12	60.53	-	-	37.50	49.31

* From rows to columns.

### Estimated hazard ratios for the effect of each exposure on BMI transition intensity

Estimated hazard ratios of each exposure factor's effect on the BMI transition intensities from the multistate model were displayed in Table 4. The results indicated that the younger (60–74) and older (≥75) elderly were associated with a 41% [HR = 0.59, 95% CI (0.46, 0.76)] and 58% [HR = 0.42, 95% CI (0.28, 0.65)] decreased likelihood of transitioning from underweight to normal BMI status, compared with the middle-aged population. In contrast, the younger and older elderly had an increased risk of shifting from normal to underweight [HR = 1.44, 95% CI (1.11, 1.86)] and overweight to normal [HR = 1.23, 95% CI (1.05, 1.44)] compared with middle-aged participants, respectively. The risk of shifting from normal BMI to overweight was 29% [HR = 1.29, 95% CI (1.13, 1.47)] greater among female relative to male. The risk of shifting from normal to underweight was 43% [HR = 1.43, 95% CI (1.07, 1.91)] higher among non-married participants compared with the married, whereas those with advanced education (middle school or beyond) had a 44% [HR = 0.56, 95% CI (0.40, 0.78)] lower likelihood of making that transition compared with whose educational attainment was elementary school or below. Those who with alcohol consumption habit had a reduced likelihood of recovering from underweight to normal [HR = 0.79, 95% CI (0.68, 0.93)] compared with the occasional drinkers (drink less than once a month); those who smoked had a decreased likelihood of transitioning from underweight to normal [HR = 0.64, 95% CI (0.50, 0.82)] and from normal to overweight [HR = 0.80, 95% CI (0.70, 0.92)] and an increased likelihood of shifting from overweight to normal [HR = 1.25, 95% CI (1.07, 1.46)] compared with the non-smokers. Participants with depression symptoms had an increased risk of transitioning from normal BMI to overweight [HR = 1.62, 95% CI (1.28, 2.04)] compared with the group without depression. Participants with ADL impairment had an higher risk of shifting from normal to overweight [HR = 1.48, 95% CI (1.06, 2.06)] compared with the participants independent in ADL, which matched most previous predictions.

**Table 4:** Estimated hazard ratios of each exposure factor effect on the BMI transition intensities by multi-state model.

	State 1^*^-State 2^†^	**State 2-State 3^§^**	**State 3-State 4^¶^**	**State 4-State 3**	**State 3-State 2**	**State 2-State 1**
**Characteristic**	**HR (95%CI)**	**HR (95%CI)**	**HR (95%CI)**	**HR (95%CI)**	**HR (95%CI)**	**HR (95%CI)**
**Age**
45–59 years	1.00	1.00	1.00	1.00	1.00	1.00
60–74 years	**0.59 (0.46, 0.76)**	**0.83 (0.73, 0.96)**	0.84 (0.68, 1.03)	1.07 (0.86, 1.34)	**1.23 (1.05, 1.44)**	**1.44 (1.11, 1.86)**
≥75 years	**0.42 (0.28, 0.65)**	0.74 (0.52, 1.04)	0.49 (0.24, 1.00)	1.08 (0.53, 2.19)	**2.02 (1.47, 2.79)**	**2.59 (1.77, 3.81)**
**Gender**
Male	1.00	1.00	1.00	1.00	1.00	1.00
Female	1.25 (0.98, 1.60)	**1.29 (1.13, 1.47)**	1.12 (0.92, 1.38)	0.92 (0.74, 1.15)	0.90 (0.77, 1.05)	1.04 (0.82, 1.32)
**Marital status**
Married with spouse present	1.00	1.00	1.00	1.00	1.00	1.00
Single, divorced or widowed	1.10 (0.82, 1.48)	0.93 (0.77, 1.12)	0.93 (0.69, 1.27)	1.26 (0.93, 1.70)	1.09 (0.88, 1.36)	**1.43 (1.07, 1.91)**
**Education level**
Illiterate	1.00	1.00	1.00	1.00	1.00	1.00
Elementary school	0.85 (0.65, 1.11)	0.97 (0.82, 1.14)	0.87 (0.68, 1.11)	1.20 (0.92, 1.55)	0.92 (0.77, 1.11)	0.77 (0.59, 1.01)
Middle school and above	0.78 (0.55, 1.12)	1.17 (0.98, 1.39)	0.86 (0.67, 1.12)	1.03 (0.78, 1.36)	0.85 (0.70, 1.04)	**0.56 (0.40, 0.78)**
**Blood pressure (mmHg)**
SBP <140 / DBP <90	1.00	1.00	1.00	1.00	1.00	1.00
SBP > 140 & DBP > 90	1.43 (0.88, 2.34)	1.24 (0.98, 1.58)	1.16 (0.87, 1.55)	0.84 (0.63, 1.12)	0.86 (0.67, 1.10)	0.70 (0.40, 1.23)
**Diabetes**
No	1.00	1.00	1.00	1.00	1.00	1.00
Yes	1.04 (0.39, 2.77)	1.18 (0.85, 1.66)	0.94 (0.63, 1.41)	1.29 (0.93, 1.78)	0.84 (0.62,1.41)	0.65 (0.30, 1.42)
**Chronic lung diseases**
No	1.00	1.00	1.00	1.00	1.00	1.00
Yes	**0.69 (0.49, 0.98)**	0.91 (0.72, 1.14)	0.80 (0.55, 1.17)	0.71 (0.46, 1.11)	1.15 (0.89, 1.48)	**1.40 (1.01, 1.95)**
**Cardiovascular diseases**
No	1.00	1.00	1.00	1.00	1.00	1.00
Yes	0.83 (0.55, 1.24)	**1.53 (1.25, 1.88)**	**1.32 (1.00, 1.73)**	0.88 (0.66, 1.17)	1.23 (0.99,1.53)	1.22 (0.84, 1.77)
**Digestive disease**
No	1.00	1.00	1.00	1.00	1.00	1.00
Yes	1.07 (0.82, 1.40)	0.88 (0.75, 1.03)	0.98 (0.77, 1.24)	1.16 (0.89, 1.49)	1.01 (0.84, 1.20)	1.27 (0.97, 1.64)
**Alcohol consumption**
Drink less than once a month	1.00	1.00	1.00	1.00	1.00	1.00
Drink more than once a month	1.12 (0.84, 1.50)	**0.79 (0.68, 0.93)**	0.91 (0.71, 1.17)	1.13 (0.86, 1.48)	1.12 (0.94, 1.33)	0.83 (0.63, 1.09)
**Smoke**
No	1.00	1.00	1.00	1.00	1.00	1.00
Yes	**0.64 (0.50, 0.82)**	**0.80 (0.70, 0.92)**	0.84 (0.68, 1.05)	1.02 (0.81, 1.29)	**1.25 (1.07, 1.46)**	1.15 (0.91, 1.45)
**Depression symptoms**
CESD-10 score <10	1.00	1.00	1.00	1.00	1.00	1.00
CESD-10 score ≥ 10	0.98 (0.77, 1.25)	1.07 (0.93, 1.22)	1.10 (0.89, 1.35)	1.09 (0.88, 1.36)	1.11 (0.95, 1.30)	**1.62 (1.28, 2.04)**
**ADL impairment**
Katz ADL Index = 12	1.00	1.00	1.00	1.00	1.00	1.00
Katz ADL Index <12	1.40 (0.83, 2.35)	**1.48 (1.06, 2.06)**	1.03 (0.60, 1.78)	0.81 (0.47, 1.40)	1.35 (0.91, 2.01)	1.15 (0.59, 2.22)

* State 1: underweight.

† State 2: normal.

§ State 3: overweight.

¶ State 4: obesity. The bold values indicate the statistically significant result.

## Discussion

Based on a nationally representative survey cohort, we found diverse longitudinal transitions of BMI status occurred among Chinese middle-aged and older adults, and the effect of exposure factors on the likelihood of BMI transition was calculated using a multistate Markov model. Our findings indicated that the likelihood of shifting from underweight to normal BMI status decreased among participants aged 60 or older, whereas the risk of transitioning from normal BMI to underweight increased among non-married participants and decreased among those with a middle school education or above (using primary school or less as the reference group indicating lower education level). Briefly, participants who were not married, less educated, and aged 60 and above were more prone to being underweight. Our findings echo previous studies that a general increase in body weight and BMI until 60 years of age, when body weight and BMI begin to decline, which could be interpreted as a change in energy intake (or absorption) or expenditure involved (12, 18). According to the predicted probability matrix, the main long-term concern for adults of normal weight should be preventing the transition to overweight rather than underweight in the middle-aged and older population. Although it was generally believed that overweight was less prevalent than undernutrition in the developing world, in fact, the prevalence of overweight in the developing world has reached an alarming state (25).

The notion that adverse lifestyle habits will lead to increase weight had been widely accepted for centuries, yet alcohol consumption more than once a month and smoking might compromise the process of weight gain but toward the body weight reduction among middle-aged and older adults. Our study found that alcohol consumption was associated with a reduced likelihood of transitioning from underweight to normal; similarly, the probability of smokers transitioning from underweight to normal and from normal to overweight decreased. Therefore, smoking might reduce the likelihood of transitioning to normal BMI. This seems to be consistent with the findings of a cohort study that noted smoking cessation was accompanied by substantial weight gain (26).

Weight loss regarded as a side effect of depression symptoms, the risk of shifting from normal BMI to underweight status increased by 62% among participants with depression symptoms in our study, which was consistent with another population-based study of adults in the United Kingdom that depressed mood was able to predict an unhealthy weight loss (27). Moreover, ADL impairment was associated with a higher risk of transitioning from normal to overweight, which matched most previous findings, among which, a study that found being overweight but not obese was associated with better daily functioning in the Colombian population (28). In summary, to move public health toward precision in public health, the specific risk factors related to transition intensity for different BMI statuses were explored to determine if personalized treatments could prevent unhealthy weight change.

A major strength of this study was its perspective cohort study design, focusing on the Chinese middle-aged and older adults used a nationally representative survey with a relatively large sample size. The high response rate (80.51%) and objective measurements guaranteed the robustness of the research results. The prospective cohort design enhanced causal inferences than cross-sectional design in observational study. Moreover, to the best of our knowledge, fitting the longitudinal data using a multistate Markov model to simulate the dynamic process of BMI status transition had not been considered in most previous studies.

Although this study included a high-quality, nationally representative sample, limitations also need to be acknowledged. First, although BMI came up as an integrated indicator to reflect the weight status, biomarkers of body composition such as body density or body fat percentage, were not available, which should assist to diagnose overweight and obesity for further research (29). Second, the generalizability of findings was limited by the potential threats of the sample selection bias. Viewing these results from a life course perspective, most of the target population in this study experienced the Great Chinese Famine in early childhood or early adulthood, with severe malnutrition affecting their adulthood body weight pattern (30). Finally, despite the mechanism underlying the weight status transition was beyond the scope of this study, our Markov process was based on the assumption that BMI changed in a relatively predictable and steady manner (31) i.e., any abrupt change across three BMI status was considered unlikely and not accounted for; thus, underestimation of transition intensity might have occurred and the effect of the co-existing risk factors time-dependent variables might be overlooked.

In conclusion, we applied a multistate Markov model to simulate the longitudinal transition of BMI status and the effect of exposure factors on the likelihood of BMI transition. The transition intensity and probability matrix indicated a common trend among Chinese middle-aged and older adults over a 4-year period. Certain risk factors including demographic characteristics (age, gender, education level, marital status), lifestyle habits (alcohol consumption, smoking), and health status (depression symptoms, ADL impairment) served as barriers or motivators of specific BMI status transitions, as clarified by the multistate Markov model.

## Data availability statement

The original contributions presented in the study are included in the article/supplementary material, further inquiries can be directed to the corresponding authors.

## Author contributions

HP performed the statistics analysis and drafted the manuscript. NK and HC participated the development of methodology and implementation of the computer code and supporting algorithms. CG and YZ helped to specifically critical review the manuscript. GC and LZ conceived of the study and participated in its design, management, and coordination and mentorship external to the core team. All authors contributed to the article and approved the submitted version.

## Funding

This study was supported by the National Key Research and Development Program of China (No. 2018YFC2000603).

## Conflict of interest

The authors declare that the research was conducted in the absence of any commercial or financial relationships that could be construed as a potential conflict of interest.

## Publisher's note

All claims expressed in this article are solely those of the authors and do not necessarily represent those of their affiliated organizations, or those of the publisher, the editors and the reviewers. Any product that may be evaluated in this article, or claim that may be made by its manufacturer, is not guaranteed or endorsed by the publisher.
